# Ultrasound-Guided Measurement of Piriformis Muscle Thickness to Diagnose Piriformis Syndrome

**DOI:** 10.3389/fneur.2021.721966

**Published:** 2021-09-07

**Authors:** Yusak Mangara Tua Siahaan, Pamela Tiffani, Amanda Tanasia

**Affiliations:** ^1^Neurology Department, Faculty of Medicine, Universitas Pelita Harapan, Tangerang, Indonesia; ^2^Neurology Department, Siloam Hospital Lippo Village, Tangerang, Indonesia

**Keywords:** diagnosis, measurement, piriformis syndrome, muscle thickness, ultrasound

## Abstract

**Background:** Piriformis syndrome (PS) is a neuromuscular condition caused by the entrapment of the sciatic nerve at the level of the piriformis muscle (PM). Diagnosing PS remains challenging despite recent invasive and non-invasive diagnostic methods. Response to invasive nerve block is still one of the most reliable diagnostic modalities because there is no gold standard test for PS. As early diagnosis may prevent delayed diagnosis that results in chronic somatic dysfunction and muscle weakness, a screening test with high sensitivity could guide clinicians in performing the next appropriate step in diagnosing PS.

**Aim:** The purpose of this study is to determine the sensitivity, specificity, and best cut-off point of ultrasound-guided PM thickness in PS.

**Method:** This case-control study was conducted in a general hospital in Tangerang during a 3-month period. We recruited 58 patients clinically diagnosed with PS and 58 healthy patients (without a history of hip and buttock pain) during their visits to the outpatient clinic. All patients underwent ultrasound assessment to measure bilateral PM thickness. Sex, age, body mass index, history of micro-/macro-trauma, and prolonged sitting duration were recorded. Statistical analyses were performed using the Statistical Package for the Social Sciences version 25.

**Result:** The PS and control groups predominantly comprised female subjects, with mean ages of 51.79 ± 14.10 and 50.09 ± 13.26 years on PS and healthy subjects, respectively. The mean ultrasound-guided PM thickness was higher in PS subjects compared to healthy subjects with mean thicknesses of 1.16 ± 0.13 and 0.85 ± 0.11 cm, respectively (*p* < 0.05). The area under the receiver operating characteristic curve of the PM was 0.970 (95% confidence interval 0.943–0.998, *p* < 0.05). The best cut-off point defined by Youden's *J* index was 0.9950 cm for all PS subjects.

**Conclusion:** We propose 0.9950 cm as the cut-off point for diagnosing PS by ultrasound, which has the sensitivity and specificity of 94.8 and 87.9%, respectively.

## Introduction

Piriformis syndrome (PS) is a neuropathic condition caused by the entrapment or irritation of the sciatic nerve by the piriformis muscle (PM). PS accounts for 6–8% of all back and sciatic pain cases (1). PM spasm may result in irritation of the nearby sciatic nerve, causing pain and numbness along the lower back of the leg to the foot (2). A study in Indonesia has found that PS mainly occurs in the fourth to sixth decade of life, especially in women, with a female-to-male ratio of 6:1 (3), and that 6% of patients with complaints of back pain were diagnosed with PS (4). The prevalence rates of PS vary from 6 to 36% (3, 4).

PS continues to be a controversial diagnosis for hip and buttock pain. To date, there has been no gold standard diagnosis for PS. Ultrasound-guided piriformis injection is an effective diagnostic modality. It not only encompasses diagnosis confirmation, where there is a response of pain relief, but also provides treatment for pelvic disorders associated with the piriformis (5). In recent years, high-resolution ultrasound (US) has been widely applied for the evaluation of entrapment neuropathies such as PS. US imaging of the PM has also been shown to act as a simple surrogate marker in the diagnosis of PS (6). US provides real-time and dynamic assessment as well as a more accessible and cost-effective option than magnetic resonance imaging (MRI) with acceptable reliability (7–9). However, this less-invasive diagnostic modality requires practitioners who are qualified and professionally skilled in performing musculoskeletal injection block, which is considered the first-choice diagnostic technique (1, 10). Early and accurate diagnosis will aid in proper management and prevent delays in diagnosis, which may result in chronic somatic dysfunction and muscle weakness. Therefore, this study evaluated the capability of US as a simple, non-invasive, and effective diagnostic method for PS by assessing the normal and abnormal ranges of PM thickness.

## Methods

This was a case-control study of 58 subjects clinically diagnosed with PS and 58 healthy subjects. All subjects who met the inclusion and exclusion criteria were collected with consecutive sampling methods. The purposive sampling was only designed in control subjects' age groups following case subjects' age group to limit the confounding effect. Ethics approval was obtained from the Institutional Clinical Research Ethics Committee of Universitas Pelita Harapan, Indonesia (no. 121/K-LKJ/ETIK/II/2021). Written consent was obtained from all the subjects before the examinations. From February 1, 2021 to April 30, 2021, we enrolled patients from the Neurology Outpatient Clinic in Siloam Hospital Lippo Village, Tangerang, Indonesia, with and without PS.

### Inclusion Criteria

The patients included in this study were aged 30 years and above. Patients meeting the following inclusion criteria were included in the PS group: patients experiencing the clinical manifestations of PS, patients with one or more positive PS physical examinations, and patients with positive diagnostic block test results [pain relief up to 75% (which is measured by a numeric rating scale) after infusion of a local anesthetic with or without corticosteroid ultrasonography-guided injection]. However, patients meeting the following criteria were included in the control group: patients with no hip or buttock pain and with normal physical examination for PS.

### Exclusion Criteria

Subjects with a body mass index (BMI) >35 kg/m^2^, surgical history involving the lumbar and/or hip region, history of buttock or hip infection, autoimmune disease, central or peripheral nervous system disorders, psychiatric diseases, and malignancy were excluded from this study. Electromyography study was not conducted in this study.

### Physical Examination

The clinical tests used to aid in the diagnosis of PS were external palpation on the piriformis line; flexion, adduction, and internal rotation test; Pace sign; Freiberg sign; and Beatty test.

### Ultrasound

**Figure 1:**
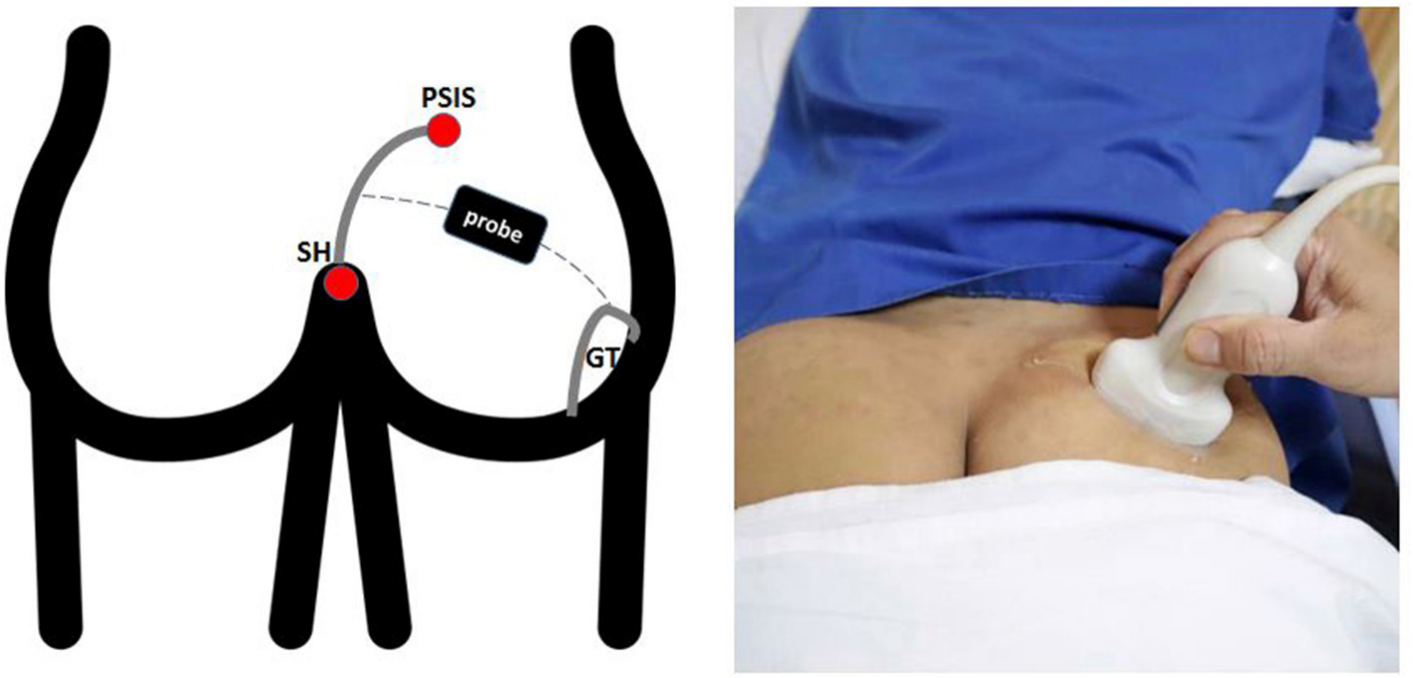
Surface landmarks. The lateral border of the sacrum was define by the line between the PSIS and the SH. The superficial line connecting the midpoint of the sacral border and the upper border of the GT runs approximately parallel to the piriformis muscle. PSIS, Posterior Superior Iliac Spine; SH, Sacral Hiatus; GT, Greater Trochanter.

**Figure 2:**
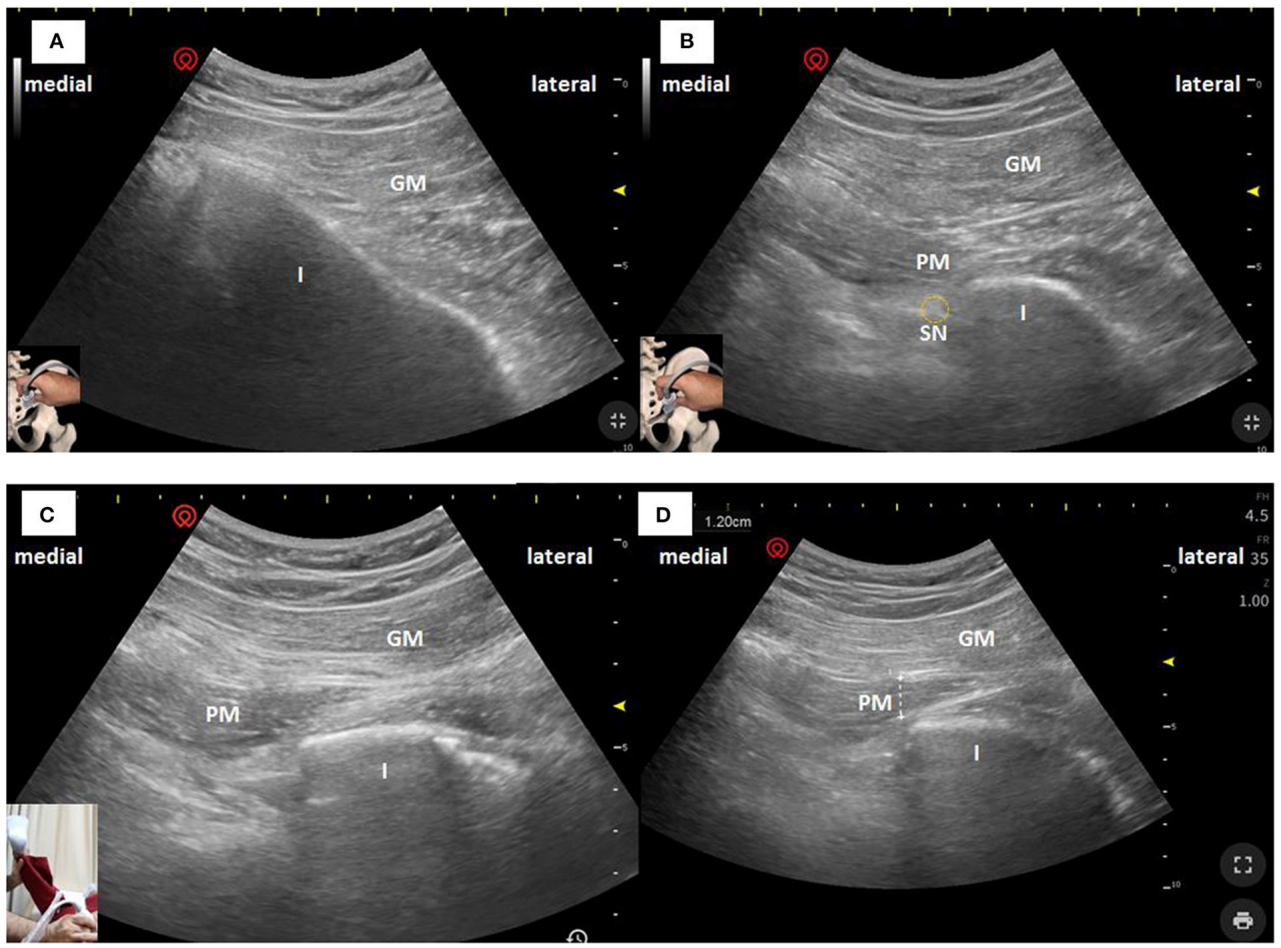
Ultrasonographic images of the PM in the longitudinal plane obtained with a curvilinear transducer at 2.5–5 MHz. The PM can be seen deeper to the GM and passing laterally toward the GT. **(A)** US image when superficial images above conducted. **(B)** US image when the clinician moved the probe inferiorly. **(C)** US image when the patient's leg was maximal abducted. **(D)** PM thickness was measured in the medial part of the tip of the ischium, which was parallel to the longitudinal plane at the sciatic notch with the patient's leg was abducted 45°. I, Ischium; GM, Gluteus Maximus; PM, Piriformis Muscle; SN, Sciatic Nerve.

PM thickness was measured using a curvilinear transducer with a 2.5- to 5-MHz bandwidth in a single US type (Wisonic Navi, WA8B30367C). All US examinations were performed by a neurologist with a certification obtained from an interventional pain sonologist. Patients were examined in the prone position, and probe was located as following Figure 1, which produced an ultrasound image as shown in Figure 2A. Then, the clinician placed the probe inferiorly to obtain Figure 2B. When ultrasound visualization was obtained, the clinician moved the patient's lower leg to maximal adduction and abduction (Figure 2C) to confirm the PM exact position. Finally, PM thickness was measured in the medial part of the tip of the ischium, which was parallel to the longitudinal plane at the sciatic notch when the patient's leg was abducted 45° (Figure 2D). With explained maneuvers, the visualization of top and bottom of the PM had clearer margin, which facilitates a simpler and easier landmark to measure. Measurement was performed thrice bilaterally.

### Statistical Analyses

Statistical analyses were performed using the Statistical Package for the Social Sciences version 25 software. Data with a normal distribution are presented as mean ± standard deviation for continuous variables and as frequencies and percentage for categorical variables. Subjects' demographic characteristics were analyzed using *t*-test for intergroup comparisons. The diagnostic performance of the significant US parameters was analyzed as the gold standard using the area under receiver operating characteristic curves (AUROCs). The AUC and 95% confidence interval (CI) were determined for both groups. The most appropriate cut-off point for diagnosis was defined using Youden's *J* index. The results were considered significant at *p* < 0.05.

## Result

A total of 58 patients clinically diagnosed with PS and 58 healthy patients were included in this study. Patients' demographic data are presented in Table 1.

**Table 1:** Demographics data of healthy and piriformis syndrome patients.

**Variables**	**Control 58**	**Case 58**	***p*-value**
Age (years)	50.09 ± 13.26	51.79 ± 14.10	0.50
Height (m)	160.34 ± 7.00	158.95 ± 7.45	0.30
Weight (kg)	64.64 ± 11.77	62.83 ± 12.78	0.43
Body mass index (kg/m^2^)	25.08 ± 3.87	24.78 ± 4.14	0.69
**Gender**			
Male	15 (51.7)	14 (48.3)	1.00
Female	43 (49.4)	44 (50.6)	
**Risk factors**			
None	9 (75)	3 (25)	0.13
≥ 1 risk factor	49 (47.1)	55 (52.9)	
**Numeric rating scale (0–10)**			
Before procedure	0	8.05 ± 1.02	0.00
After procedure	0	1.98 ± 0.80	

There were no significant differences in age, weight, height, BMI, sex, and risk factors [history of micro-/macro-trauma, prolonged sitting duration (>6 h/day)] between case and control subjects. Meanwhile, the mean of PM thickness in the symptomatic case group was higher than that in the control group (*p* < 0.05; Table 2).

**Table 2:** Mean and standard deviations of the PM thickness.

**PM Thickness (cm)**	**Control (58 Healthy)**			**Case (58 PS)**		
	**Right**	**Left**	***P*-value**	**Asymptomatic**	**Symptomatic**	***P*-value**
Mean ± SD	0.86 ± 0.11	0.85 ± 0.11	0.38	0.89 ± 0.11	1.16 ± 0.13	0.00^*^

* *p value < 0.05 was considered significant*.

**Figure 3:**
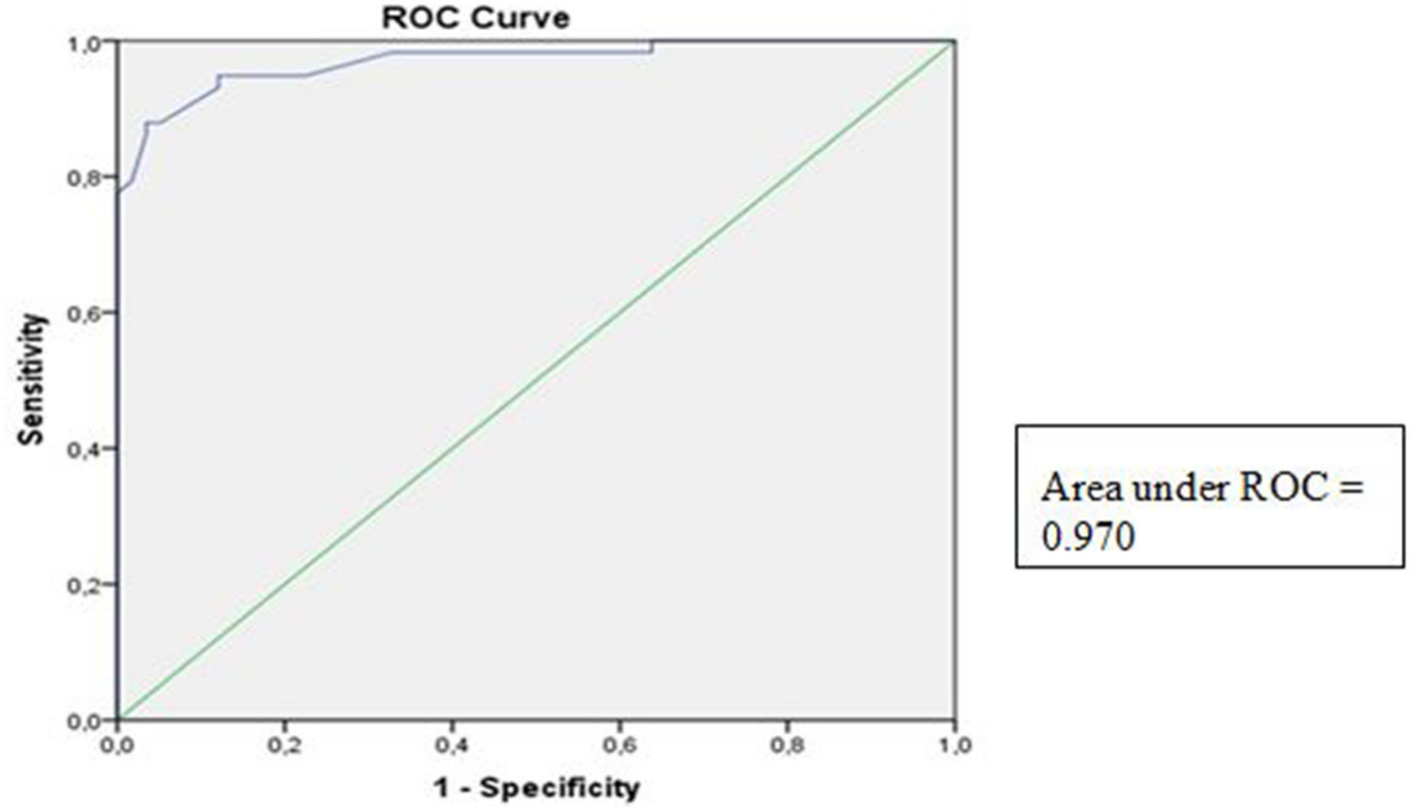
Receiver operator curve for piriformis muscle thickness.

ROC analyses were performed on PM thickness between the PS and healthy subjects. The AUROC for the symptomatic case group was 0.970, with a 95% CI of 0.943–0.998 (*p* < 0.05) (Figure 3).

Using the Youden index method (*J*), the optimal cut-off point of PM thickness (centimeters) for identifying PS was 0.9950 cm (Table 3).

**Table 3:** Diagnostic value of various cut-off point of muscle thickness in determining PS.

**Muscle thickness**	**Sensitivity**	**Specificity**	**Accuracy**	**LR+**	**Youden Index**
0.9700	94.8	86.2	90.5	6.87	0.81
0.9950^*^	**94.8**	**87.9**	**91.4**	**7.83**	**0.827**
1.0100	93.1	87.9	93.9	7.69	0.81

* *Best cut-off point*.

## Discussion

To date, various modalities have been used to diagnose PS, including computed tomography (CT), MRI, electromyography (EMG), and US. However, all these methods have both advantages and disadvantages for diagnosing PS. Although EMG may indicate nerve damage and assist in establishing differential diagnosis, its main disadvantage is its low specificity and poor repeatability, leading to difficulty in evaluating the lesion site. Moreover, CT is unable to provide sufficiently clear images to visualize subtle changes in muscles and soft tissues. In addition, MRI may not be practical for routine use in the management and risk assessment of patients because of its long imaging time, high cost, and difficulty in nerve reconstruction (7). Therefore, US is superior among other available image-guided modalities in terms of reliability, simplicity, accessibility, cost-effectiveness, availability at the bedside, and absence of ionizing radiation over CT or MRI (5, 7, 11, 21).

Moreover, few studies have shown that US measurement of muscle thickness is reliable for musculoskeletal pathology diagnosis, such as in patients with supraspinatus tendon pathology by Kretić et al. (12) and thigh muscle thickness for the assessment of sarcopenia by Hida et al. (13). Hence, we suggest that the US-guided measurement of PM thickness is one of the major diagnostic criteria for PS. Previous studies have shown different measurement components (1, 7, 10, 14–16). In the study by Demirel, PS was diagnosed by increasing muscle elasticity and tissue hardening with US elastography (1). However, this preliminary study had a relatively small number of cases and has not yet been established in many studies. Several studies applied PM thickness measurement with other components such as echogenicity and dynamic signs (14), cross-sectional area of the muscle (6), and sciatic nerve diameter (7). The measurement of the cross-section length of the sciatic nerve is difficult to be assessed at the level of PM, because of its deep location and absence of obvious adjacent landmarks (16). Other studies showed ultrasound-guided posterior approaches to the sciatic nerve indicated a position slightly distal to the subgluteal fold as an advantageous position in terms of superficial nerve position and good ultrasonographic visibility (17). Therefore, the association between PS and sciatic nerve sizes cannot be determined in this study.

Our study found that the mean PM thickness in PS patients was significantly higher compared to the healthy subjects (1.16 ± 0.13 and 0.89 ± 0.11, respectively, *p* = 0.00). These results are similar to those of Zhang et al. (10), Todorov et al. (14), and Wu et al. (7) who found increased mean PM thickness in PS subjects. Although the study by Siddiq et al. had the same results, the PM thickness difference in those study was not statistically significant (15). The pathophysiology of PM enlargement is still unclear, but several hypotheses have mentioned that PS occurs due to single blunt trauma or macro-trauma and long-term microtrauma causing PM spasm, inflammation, and hypertrophy (10, 18, 19).

Apart from the significance of PM thickness in US, previous studies have also shown a wide variation in PM thickness in their study without detailed US methods. In the study of Todorov et al. (14) the anterior-posterior PM thicknesses of PS were 5.8 to 11.55 mm (males) and 4.4 to 9.6 mm (females), whereas the anterior-posterior PM of PS was 13.55 ± 3.66 mm (affected) in the study of Siddiq et al. (15). Those variations might be caused by the subject's different activities. This statement is also supported by Park et al. who found that PM stretching methods are effective in reducing PM thickness (20). Specifically, our study also had different variations from previous studies because of the different methods applied.

To the best of our knowledge, only an in-review study had detailed US methods to measure PM thickness using the maximum anteroposterior diameter (MAPD) mean value of the long-axis view and short-axis view. The MAPD was measured along the lateral border of the epimysium at the thickest muscle segment in the long-axis and short-axis views of the piriformis (7). However, these methods require more complicated techniques and require more time to identify the overall PM anatomically. Therefore, our study proposes a simpler and more efficient way to measure PM thickness. This study used the medial part of the tip of the ischium to visualize the PM that was parallel to the longitudinal plane at the sciatic notch while the patient's leg was abducted at the 45° position. This method has clearer boundaries for viewing the PM, thus making the test more reproducible.

According to the literature, several methods have been described and studied to identify PM thickness. However, studies assessing the cut-off and validity standard of PM thickness to track PS condition have not been conducted (10–14). In this study, we found that the AUROC of PM thickness was 0.970 (95% CI 0.943–0.998, *p* < 0.05), suggesting that the PM thickness was a good predictor of PS. This study found that the best PM thickness cut-off point to diagnose PS according to Youden's *J* index was 0.9950 cm, with a sensitivity of 94.8% and a specificity of 87.9%. These results suggest that PM thickness by US may be ideal to establish the diagnosis of PS.

## Limitation of the Study

Although PM thickness varies widely in other studies, it might be considered a limitation despite any contribution of different activities or races. Further studies comprising large sample sizes, assessing multifactorial causes, and comparing US with other imaging modalities are needed.

## Conclusion

In conclusion, PM thickness measurement by US may be a reliable technique for the early diagnosis of PS, increasing the possibility of conservative treatment methods, and may also be used to routinely evaluate patients with unidentified causes of buttock pain. We propose 0.9950 as the cut-off point for diagnosing PS by US, which has a sensitivity and specificity of 94.8 and 87.9%, respectively.

## Data Availability Statement

The raw data supporting the conclusions of this article will be made available by the authors, without undue reservation.

## Ethics Statement

The studies involving human participants were reviewed and approved by Universitas Pelita Harapan. The patients/participants provided their written informed consent to participate in this study.

## Author Contributions

All authors contributed to the design and implementation of this study, to the analysis of the result, and to the writing of manuscript.

## Funding

The funding for this research was financed independently by authors.

## Conflict of Interest

The authors declare that the research was conducted in the absence of any commercial or financial relationships that could be construed as a potential conflict of interest.

## Publisher's Note

All claims expressed in this article are solely those of the authors and do not necessarily represent those of their affiliated organizations, or those of the publisher, the editors and the reviewers. Any product that may be evaluated in this article, or claim that may be made by its manufacturer, is not guaranteed or endorsed by the publisher.
